# Comparing Effectiveness and Safety of Left Atrial Appendage Closure Devices: A Network Meta‐Analysis of Randomized Controlled Trials

**DOI:** 10.1002/clc.70217

**Published:** 2025-11-22

**Authors:** John W. Davis, Steven L. Mai, Wissam Harmouch, Jenna Reisler, Micaela MacKay, Elizabeth Davis, Pavel Osmancik, Michael W. Rich

**Affiliations:** ^1^ Department of Internal Medicine Washington University in St. Louis/Barnes‐Jewish Hospital St. Louis Missouri USA; ^2^ Department of Internal Medicine University of Texas Medical Branch at Galveston Galveston Texas USA; ^3^ Department of Internal Medicine, Division of Cardiovascular Medicine University of Texas Medical Branch at Galveston Galveston Texas USA; ^4^ University Hospital Kralovske Vinohrady Charles University Prague Czechia; ^5^ Cardiovascular Division Washington University in St. Louis St. Louis Missouri USA

## Abstract

**Introduction:**

Atrial fibrillation‐related stroke is a leading cause of morbidity and mortality. The comparative effectiveness and safety of left atrial appendage closure (LAAC) devices, compared with one another and with anticoagulation, is unclear.

**Methods:**

We conducted a systematic review and network meta‐analysis (NMA) of all clinical trials comparing the Watchman and Amplatzer Amulet LAAC devices with each other or with warfarin or direct oral anticoagulants (DOACs). The primary comparison was between LAAC devices with secondary comparisons to anticoagulation. The primary effectiveness outcomes were any stroke and all‐cause death. Safety outcomes included any thromboembolism, device embolization, and pericardial effusion.

**Results:**

There were 476 articles identified from the search and 6 eligible RCTs were included (*n* = 3666). There was no difference in the risk of stroke with Amulet versus Watchman (RR = 1.48, 95% CI: 0.64–3.46, *I* ^2^ = 41.3%), nor in the risk of death (RR = 1.00, 95% CI: 0.59–1.70, *I* ^2^ = 45.0%). Risk of thromboembolism was not significantly different with Amulet versus Watchman (RR = 0.73, 95% CI: 0.18–2.97, *I* ^2^ = 0%), nor was risk of device embolization (RR = 2.29, 95% CI: 0.71, 7.43, *I* ^2^ = 0%). Both devices exhibited increased risk of pericardial effusion compared with warfarin, with Amulet at highest relative risk (RR = 27.08, 95% CI: 3.53–207.98, *I* ^2^ = 0%) followed by Watchman (RR = 12.79, 95% CI: 1.73–94.85, *I* ^2^ = 0%). Amulet also carried higher relative risk of pericardial effusion than Watchman (RR = 2.12, 95% CI: 1.45–3.09).

**Conclusion:**

In this NMA, the Amulet and Watchman LAAC devices were associated with similar risks for stroke, mortality, thromboembolism, and device embolization. Pericardial effusion risk was higher with Amulet than Watchman.

## Graphical abstract

In this network meta‐analysis of randomized controlled trials comparing Watchman, Amplatzer Amulet, and/or anticoagulation, we found evidence that Amulet was associated with significantly greater risk of pericardial effusion than Watchman (RR = 2.12). Safety and effectiveness of these devices were otherwise comparatively similar.

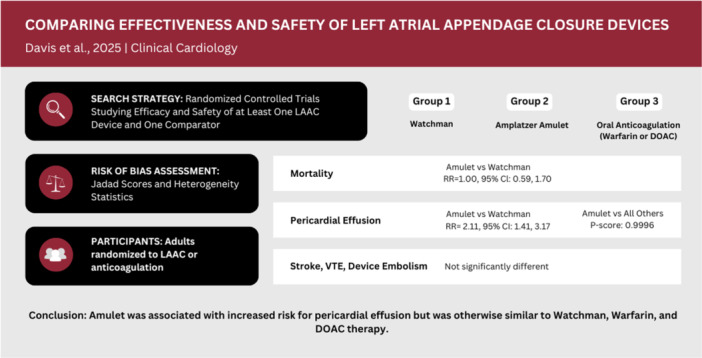

## 1 Introduction

Despite advances in management, stroke is the second leading cause of death and the third leading cause of disability worldwide [1]. Atrial fibrillation (AF) is the most common underlying cause of cardioembolic stroke and increases an individual's risk of stroke fivefold [2]. The left atrial appendage (LAA) is a common source of emboli that has been implicated in approximately 90% of AF‐related stroke [2]. Compared with strokes secondary to carotid disease, strokes related to LAA thrombi are associated with greater disability burden and higher all‐cause mortality [3].

Although oral anticoagulation (OAC) effectively reduces the risk of stroke and thromboembolism among patients with AF [4], these agents require medication adherence and are associated with bleeding complications that constitute the main reason for OAC discontinuation during long‐term management [5]. Thus, percutaneous left atrial appendage closure (LAAC) devices have been developed to prevent thrombus embolization and cardioembolic stroke without the need for long‐term OAC [6]. The Watchman device, a single‐seal LAAC device approved by the FDA in 2015, prevents thromboembolic events with effectiveness noninferior to warfarin [6]. The Amplatzer Amulet (Amulet) device was approved for LAAC in 2021 after demonstrating noninferiority in preventing ischemic stroke and systemic embolism when compared with Watchman [6]. Compared with Watchman, the mechanism of LAA occlusion differs with the Amulet. The device consists of two parts, with the distal lobe fixed deeper in the LAA and the LAA ostium covered by the proximal disc. This allows more ostial LAA closure and less peri‐device leakage [6].

Primary safety analysis from the Amulet Investigational Device Exemption (IDE) trial demonstrated higher rates of procedural‐related complications, such as pericardial effusions and device embolization, when compared with Watchman despite a higher success rate of complete LAA closure [6]. However, this and other trials were underpowered for stroke and mortality. A network meta‐analysis (NMA) utilizes both direct and indirect estimates from included studies to increase power for detecting small but important associations. The present NMA aims to investigate the comparative effectiveness and safety of LAAC devices, both head‐to‐head and against OAC.

## 2 Methods

### 2.1 The Trials

PubMed, Cochrane Database, and Web of Science were searched for systematic reviews, meta‐analyses, and/or randomized clinical trials published in English on Watchman and/or Amplatzer Amulet devices. The search dates were from 1946 to January 1, 2025. Methods were registered prospectively with Prospero (CRD# 42023428226; see Supporting Information for search terms and strategy). We aimed to include all RCTs comparing either device to anticoagulation (either warfarin or direct oral anticoagulants, DOACs) or to the alternative device for subjects with AF. There were no additional exclusion criteria for trial eligibility. At least two authors blinded to codification by other reviewers identified trials for final inclusion, then coded each for quality with a 5‐point Jadad quality score [7]. Data on exposure, outcomes, comorbidities, and trial design were also extracted and collated for analysis. We also searched clinicaltrials.gov for any additional information, and reported whichever source was more comprehensive for each outcome. After independent extraction, any disagreements were arbitrated by the principal investigator (J. W. D.). We made attempts to contact trial investigators for clarifications when needed.

### 2.2 Exposure Variable

Study arms were classified as “Watchman‐exposed,” “Amulet‐exposed,” “warfarin‐exposed,” or “DOAC‐exposed.”

### 2.3 Outcome Variables

We first aimed to code for effectiveness of the intervention—prevention of stroke (either ischemic, embolic, or hemorrhagic) and/or death. Adjudication as intervention‐related or unrelated was ignored for all outcomes to avoid bias and because of variable event definitions across trials. Trial definitions for each outcome were used because the data were study‐level and could not be reliably abstracted otherwise.

The primary effectiveness outcome was any stroke (ischemic or hemorrhagic). The secondary effectiveness outcome was death from any cause. We next assessed adverse event outcomes, including thromboembolism, device embolization, and pericardial effusion [8, 9]. All events were counted regardless of whether trialists identified the event as “intervention‐related.”

### 2.4 Analysis

We utilized standard methods for conducting network meta‐analyses [10]. Published aggregate data from each trial were used, except for PRAGUE‐17, which required unpublished data for analysis. Prevalence estimates were calculated for each outcome by intervention arm. Pooled relative risk (RR) was estimated for each outcome, as follow‐up time was approximately equal across each intrastudy arm. Since some events, such as device embolization, are not possible with OAC controls, we used a 0.5 continuity correction to generate RRs where 0 cells were present.

When conducting the NMA, we utilized the Inverse Variance method with random effects in the “netmeta” package [11] to generate risk ratios for each outcome. We assessed the percentage of evidence utilized for each comparison that is “indirect” (comparing interventions across trials) for each outcome. Risk of bias related to heterogeneity was assessed for all trials using standard measures (*I* ^2^, Cochran's *Q*) [12, 13] in addition to generating funnel plots. High risk of heterogeneity bias was defined as an *I* ^2^ point estimate ≥ 75% [12, 13]. Because there was a small number of eligible trials, meta‐regression and formal hypothesis tests for bias were not feasible. We qualitatively assessed the transitivity assumption—that there is no effect modification with baseline characteristics of subjects across trials—by confirming that age, comorbidities, and AF‐related stroke risk were approximately equal at baseline. League tables providing RR estimates of all available comparisons were generated to display multiple comparisons.

Because NMA indirect evidence breaks randomization, causal language may not be appropriate [14]. We therefore avoid doing so here. However, we used a frequentist tool (“netrank” command in the netmeta package) [11, 15] to quantify the certainty that one intervention was superior to alternatives when at least one comparison was significantly different. A “*P*‐score,” the inverse of a one‐sided hypothesis test of superiority, was estimated for each outcome. A *P*‐score of < 0.05 suggests there is less than a 5% average certainty that the intervention is superior to alternatives, whereas a *P*‐score > 0.95 indicates at least 95% certainty the intervention is superior to alternatives [15]. For ease of interpretation, we report inverse *P*‐score (the certainty of inferiority) where one intervention appears worse than alternatives.

We followed PRISMA guidelines for reporting and analyzing these studies. All analyses were conducted in R, Version 4.1.3 [16].

### 2.5 Ethics and Data Availability

This meta‐analysis of aggregated data is exempt from IRB review. Data and code used for analysis are available on reasonable request.

## 3 Results

### 3.1 Search Results

As of January 1, 2025, there were 476 articles that were identified for review, of which 34 (7.1%) were either RCTs, systematic reviews, or meta‐analyses that contained potentially eligible trials. From these, we identified five RCTs [8, 9, 17, 18, 19] that met criteria, and data were abstracted for analysis. The PRAGUE‐17 [20] trial was also identified as potentially eligible for inclusion, but data for Amulet versus Watchman were not reported. We contacted the principal investigator (P. O.) to obtain unpublished data assessing LAAC devices separately. Thus, we included six total RCTs in the NMA. Three trials [9, 18, 19, 21] assessed both LAAC devices head‐to‐head, PRAGUE‐17 randomized participants to either LAAC device or anticoagulation with a DOAC, and the other two trials [8, 17] compared Watchman with warfarin. The PRISMA diagram (Supporting Information S1: eFigure 1) displays the search study flow.

### 3.2 Study Quality and Subjects Enrolled

Summaries of study quality and characteristics are found in Table 1. Jadad scores for study quality varied. Because subjects and healthcare providers could not be blinded to Watchman versus OAC, the two earliest trials [8, 17] scored 3/5 for study quality. The PRAGUE‐17 trial [20] scored 4/5 for blinding and randomizing to LAAC or DOAC, but by design did not randomize to Watchman versus Amulet. Two [9, 18] additional trials scored 4/5 possible points (Lakkireddy [9], lack of echo technician blinding; Mansour [18], time sequence‐based randomization), and one trial [19] scored 5/5 points for quality. Of note, the two trials assessing Watchman versus OAC excluded subjects with contraindications to warfarin, whereas the four remaining trials did not. There were no other apparent differences in baseline comorbidities across interventions.

**Table 1:** Descriptive statistics.

Author, year (trial name)	Comparison groups	*N*	N Watchman	N Amulet OR Warfarin	N DOAC	Gen.Watch.	Warfarin exclusion	Age (mean)	Female (%)	Mean F/u (days)	Jadad score
Osmancik 2022 (Prague‐17)	Watchman, Amulet, DOAC	380	70	111	199	2.5/FLX	N	73.4	33.3	1278	4
Galea 2022 (SWISS‐APERO)	Watchman versus Amulet	221	110	111	NA	2.5/FLX	Y	76.9	29.4	45	5
Lakireddy 2021, 2024 (Amulet IDE)	Watchman versus Amulet	1878	944	934	NA	1st Gen.	N^a^	75	40	540	4
Mansour 2021	Watchman versus Amulet	51	25	26	NA	2.5/FLX	N	75.5	23.5	365	4
Holmes 2014 (PREVAIL)	Watchman versus Warfarin	407	269	138	NA	1st Gen.	Y	74.3	30	540	3
Holmes 2009 (PROTECT AF)	Watchman versus Warfarin	707	463	244	NA	1st Gen.	Y	72	29.7	540	3

a Long term OAC Contraindication, but considered safe for short‐term warfarin therapy.

Of the subjects included, 1881 (51.6%) were randomized to Watchman, 1182 (32.4%) were randomized to Amulet, 382 (10.5%) were randomized to warfarin, and 199 (5.5%) were randomized to DOACs (*N* = 3644 subjects overall). Average age for participants across interventions ranged from 71.7 to 76.5 years, the average Congestive heart failure, Hypertension, Age ≥ 75 (2 points), Diabetes mellitus, prior Stroke or transient ischemic attack (2 points), Vascular disease, Age 65–74, Sex female (CHA_2_DS_2_‐VASc) score ranged from 2.0 to 2.8 [22], and the average Hypertension, Abnormal renal/liver function, Stroke, Bleeding history or predisposition, Labile INR, Elderly, Drugs/alcohol concomitantly (HAS‐BLED) [23] score (among the three head‐to‐head trials) ranged from 3.1 to 4.1. Follow‐up time ranged from 45 days [18] to 3.5 years [20]. The full descriptive statistics are displayed in Table 1.

Because we used previously unpublished data from the PRAGUE‐17 trial, we present these data in the Supporting Information. The descriptive statistics for this trial can be found in Supporting Information S1: eTable 1. There were 199 participants randomized to DOAC therapy and 181 randomized to LAAC. Of these, 111 received Amulet implantation and 70 received Watchman at the discretion of the treating physician. However, despite not separately randomizing participants to Amulet or Watchman, all intervention groups in PRAGUE‐17 were similar in age, CHA_2_DS_2_‐VASc score, HAS‐BLED score, and prior anticoagulation history. Event rates for all outcomes prespecified for these analyses, in addition to other unpublished but relevant outcomes, are displayed in Supporting Information S1: eTable 2. Participants who received Watchman had nonsignificantly lower rates of stroke and death and no thromboembolic events, pericardial effusions, or device embolizations. The estimated absolute risk differences for these outcomes versus DOACs or Amulet were generally low.

**Table 2:** Effectiveness network meta‐analysis league table.

Relative risks of the primary efficacy endpoint by treatment group						
	Risk of stroke: RR [95% CI]			Risk of death: RR [95% CI]		
	**Versus Watchman**	**Versus DOAC**	**Versus Warfarin**	**Versus Watchman**	**Versus DOAC**	**Versus Warfarin**
Amulet	1.48 [0.64; 3.46]	1.40 [0.40; 4.85]	1.36 [0.40; 4.71]	1.00 [0.59; 1.70]	0.79 [0.39; 1.59]	0.69 [0.31; 1.53]
Watchman	—	0.94 [0.23; 3.92]	0.92 [0.37; 2.28]	—	0.79 [0.37; 1.67]	0.69 [0.38; 1.25]
DOAC	—	—	0.98 [0.18; 5.28]	—	—	0.89 [0.34; 2.29]

### 3.3 Effectiveness Outcomes

All six eligible trials reported the risk of any stroke and were included in this analysis. The forest plots and *P*‐scores for all outcomes can be found in the Supporting Information (Supporting Information S1: eFigures 2–6). The primary effectiveness outcome results are displayed in Table 2. The observed risk of any stroke, when comparing Amulet versus Watchman, was not different (RR = 1.48, 95% CI: 0.64, 3.46). The overall *I* ^2^ was 41.3% (Cochran's *Q p* = 0.15), indicating moderate risk of heterogeneity across trials. Comparisons of devices to OAC with respect to stroke risk were nonsignificant.

Funnel plots suggested the Amulet IDE trial may be an outlier (Supporting Information S1: eFigure 7). When excluding this trial, estimates suggested a trend to increased risk of stroke with Amulet versus Watchman (RR = 3.22, 95% CI: 0.89, 11.67, *p* = 0.076). Heterogeneity bias risk estimates decreased with exclusion of this trial (*I* ^2^ = 12.2%).

All six trials also reported all‐cause death and were included. The risk of death from any cause was not different across any comparison group. Head‐to‐head, risk of death with Amulet was estimated to be nearly identical with that of Watchman (RR = 1.00, 95% CI: 0.59, 1.70). The *I* ^2^ estimate was 45.0% (Cochran's *Q p* = 0.12), indicating moderate risk of heterogeneity. There were again no differences between LAAC devices and warfarin or DOACs.

As with stroke, the Amulet IDE trial was a possible outlier (Supporting Information S1: eFigure 8). When excluding this trial, the risk of death was nonsignificantly higher with Amulet versus Watchman (RR = 1.57, 95% CI: 0.87; 2.86). The *P*‐score for Watchman was 0.953, indicating an approximately 95% probability that Watchman was the superior treatment option of all those assessed.

### 3.4 Safety Outcomes

#### 3.4.1 Thromboembolism

The safety outcome results are displayed in Table 3. Five of the six trials reported the risk of any thromboembolic event and were included. Risk of thromboembolism with Amulet was not different than with Watchman (RR = 0.73, 95% CI: 0.18, 2.97). There were also no significant differences compared with warfarin or DOACs. Risk of heterogeneity bias was estimated to be low (*I* ^2^ = 0%, Cochran's *Q p* = 0.99). Funnel plots were not suggestive of outlier influence (Supporting Information S1: eFigure 9).

**Table 3:** Safety network meta‐analysis league table.

(A) Relative risks of thromboembolic events							
	Risk of thromboembolism: RR [95% CI]					Risk of device embolization: RR [95% CI]	
	**Versus Watchman**		**Versus DOAC**		**Versus Warfarin**	**Versus Watchman**	
Amulet	0.73 [0.18; 2.97]		0.64 [0.05; 8.80]		1.50 [0.11; 20.32]	2.29 [0.71; 7.43]	
Watchman	—		0.88 [0.06; 12.00]		2.04 [0.23; 18.44]	—	
DOAC	—		—		2.33 [0.08; 71.23]	—	

**(B) Relative risks of pericardial effusion**							
		**Risk of pericardial effusion: RR [95% CI]**					
		**Versus Watchman**		**Versus DOAC**			**Versus Warfarin** a
Amulet		2.12 [1.45; 3.09]*		—			27.08 [3.53; 207.98]**
Watchman		—		—			12.79 [1.73; 94.85]***
DOAC^a^		—		—			—

a 0 Events in DOAC and warfarin groups across studies; 0.5 continuity correction used for warfarin comparator.

* *p* = 0.001

** *p* = 0.002

*** *p* = 0.01.

#### 3.4.2 Device Embolization

Because the risk of device embolization is nonexistent with OACs, we report only head‐to‐head risk ratios for this outcome. Four trials reported this event and were analyzed. Risk of device embolization was not significantly different between Amulet and Watchman (RR = 2.29, 95% CI: 0.71, 7.43). Estimated risk of heterogeneity bias was low (*I* ^2^ = 0%, Cochran's *Q p* = 0.97). Funnel plots were again unremarkable (Supporting Information S1: eFigure 10).

#### 3.4.3 Pericardial Effusion (With or Without Tamponade)

Pericardial effusion, with or without tamponade, was reported in 5/6 trials. Trials varied in their reporting of effusions. For example, some subcategorized effusions as “serious” versus “non‐serious,” “with tamponade” or “without tamponade,” and “treatment‐related” or “treatment‐unrelated.” Here, we report all cumulative event counts for each group.

When compared with warfarin, the risk of any pericardial effusion was significantly greater with both Amulet (RR = 27.0, 95% CI: 3.53, 208) and Watchman (RR = 12.8, 95% CI: 1.73, 94.9). Head‐to‐head, risk of effusion was over two‐fold greater with Amulet than with Watchman (RR = 2.12, 95% CI: 1.45, 3.09). The *P*‐score suggested that the certainty of increased risk with Amulet compared with alternatives was greater than 99% (Inverse *P*‐score = 0.9996), and the certainty that warfarin carried less risk was likewise greater than 99% (*P*‐score = 0.997). Risk of pericardial effusion was not different between DOACs and warfarin (0 events in each group). Risk of heterogeneity bias was again estimated to be low (*I* ^2^ = 0%, Cochran's *Q p* = 0.52), and funnel plots revealed no meaningful deviations across trials (Supporting Information S1: eFigure 11).

#### 3.4.4 Sensitivity Analysis

Because there was concern for potential bias related to a “learning curve” phenomenon during the initial Watchman noninferiority trials (i.e., vs. OACs), a sensitivity analysis was performed excluding these trials. Although estimates were similar to the main analysis, they were likely underpowered due to smaller sample size. For example, relative risk estimates of stroke with Amulet versus Watchman were similar (RR = 1.79 vs. 1.48, both nonsignificant).

## 4 Discussion

This comprehensive network meta‐analysis of RCTs examining the effectiveness and safety of LAAC devices provides important evidence on the potential differences between Watchman and Amulet. While both Watchman and Amulet serve the purpose of occluding the LAA, differences in design lead to differences in deployment protocols that may impact effectiveness and safety. Previous meta‐analyses were either underpowered (lacking utilization of indirect evidence) or included nonrandomized cohort studies. In contrast, our analysis includes data from all relevant comparative RCTs, as well as previously unpublished data from the PRAGUE‐17 trial.

The principal findings of our analysis are that the Amulet and Watchman LAAC devices are associated with similar effectiveness with respect to all‐cause stroke and all‐cause mortality. Pericardial effusions of any severity were more common with either device than with OAC, and more common with Amulet than with Watchman. However, the absolute number of effusions was small, and the apparent difference in effusions could reflect differences between trials in reporting of effusions, operator experience, or chance related to low event rates. Other complications, including thromboembolism and device embolization, did not differ between Amulet and Watchman. Hence, while we cannot completely exclude differences between devices, our findings are generally reassuring that there are no major differences in effectiveness or safety between currently available LAAC closure devices. Of note, our findings are also consistent with recently published real‐world data [24].

The Amulet IDE trial [21] was identified as a possible outlier in both effectiveness outcome analyses, and its exclusion resulted in lower heterogeneity risk bias estimates. The significance of this is unclear. The trial was sponsored by the device manufacturer, which was responsible for site and “implanter” selection, trained all implanters in use of Amulet, and allowed up to three “roll‐in” cases that were not included in analysis. However, the steering committee and investigators had complete access to the data, and individual proceduralists functioned independently of the sponsor. Additional analyses utilizing individual patient data are necessary to further evaluate the comparative efficacy of Amulet and Watchman devices.

In this study, we used NMA to increase the statistical power to detect differences between interventions for underpowered outcomes [15]. Death was primarily a “safety” endpoint in these trials (though often included in efficacy composite endpoints), and it occurred relatively infrequently, in part due to the short duration of the trials (18 months or less in all trials except PRAGUE‐17). Results from the Amulet IDE suggest that absolute rates of successful deployment—defined as no significant leak postprocedure—were approximately 2.1% higher with Amulet compared with Watchman [6]. However, the difference in rate of successful deployment did not appear to translate to decreased risk of stroke or all‐cause death. The AMULET IDE trial also demonstrated that the Amulet was noninferior to Watchman for prevention of stroke and systemic embolism [6].

We also present previously unpublished, de‐aggregated data from the PRAGUE‐17 trial, one of the largest LAAC trials to date. We did not perform ad‐hoc hypothesis tests on these data because patients were not randomized to receive Amulet or Watchman, and the sample sizes were too small to adjust for potential confounders. There was no obvious selection bias between groups: participants who underwent LAAC had similar baseline characteristics regardless of the chosen device, and the hazard ratio for the primary endpoint, as well as for secondary endpoints, including stroke and bleeding, did not differ between centers. Because the reasons for selecting one device over the other are unclear, caution should be taken in interpreting these nonrandomized comparative data.

Although the Amulet IDE trial demonstrated higher rates of successful deployment and similar effectiveness with Amulet compared with Watchman, procedural complications—primarily pericardial effusion and device embolization—were more common with Amulet (4.5% vs. 2.5%) [6]. While the absolute risk is low, our NMA demonstrated a two‐fold greater risk of pericardial effusion with Amulet than Watchman (RR = 2.11). In the PRAGUE‐17 trial [20], two events occurred in the Amulet group and none with Watchman; both of these pericardial effusions were late (> 80 days postprocedure) and one required pericardiocentesis. The mechanisms for the possible increased risk of pericardial effusions with the Amulet device are unclear but could include variability in definitions and reporting of effusions across trials and operator experience. In addition, the Amulet, once deployed, typically has greater surface contact with the LAA, which may result in inflammation and pericardial effusion [6]. The risk appears to be exacerbated by operator inexperience, as suggested by the Amulet IDE trial, in which no effusions occurred in subjects undergoing implantation by an interventionalist with > 9 cases of experience [6].

### 4.1 Limitations

This study has some limitations. As previously noted, NMAs use all available evidence to generate effect estimates for each intervention. Because these estimates are nonrandomized, we refrain from interpreting these results causally. A prior direct meta‐analysis [25] compared Watchman with Amulet, but 16 of 19 included studies were observational, thus increasing the risk for bias and confounding. In addition, this study did not include indirect RCT evidence and thus had more limited statistical power from RCTs to evaluate rarer outcomes such as death.

Warfarin, which is highly effective in preventing AF‐related ischemic stroke [26] but has an unfavorable safety profile compared with Factor Xa inhibitors (DOACs), was the primary anticoagulant studied in these analyses [27]. This limits the interpretation of comparative effectiveness and safety of OACs, but does not impact the device comparisons. Our study also combined the original, the 2.5, and the FLX generations of Watchman devices for comparison with the Amulet LAAC device and OACs. Newer generations of both Amulet and Watchman devices have since been released, which may differ in safety and effectiveness when compared with previous versions. However, one included study [18] suggested Watchman 2.5 and FLX were not meaningfully different in efficacy and safety outcomes and therefore might reasonably be combined. In contrast, a more recent retrospective cohort analysis suggested Watchman FLX may be associated with lower risk of device leak and stroke [28] compared with earlier device generations. Further subcategorization by generation would be preferable, but there is insufficient power for such analyses. Therefore, it is unclear whether Watchman FLX would offer better comparative effectiveness and safety versus Amulet than estimates generated in this study. Exclusion of the two earliest trials (Watchman vs. warfarin) did not change the estimate for any of the prespecified outcomes in Amulet versus Watchman.

Study‐level differences in (1) exclusion criteria, (2) operator experience, (3) event definitions, and (4) postimplantation antiplatelet/anti‐coagulation strategies could have impacted event rates and confounded our findings. For example, the exclusion of subjects with a prior history of major bleeding in the studies where subjects were randomized to warfarin or Watchman [8, 17] likely decreased the absolute risk of death and hemorrhagic stroke within the trials, while inclusion of inexperienced operators in the Amulet‐IDE trial may have increased the risk of pericardial effusion [24]. While the first issue might limit power by reducing event rates, it is less likely to change relative risk estimates. The second issue suggests that adverse event rates attributable to the procedure in these trials may be most generalizable to settings where proceduralists have limited experience with LAA closure. The problem of differences in event definitions across trials was mitigated by our decision not to distinguish, for example, between “treatment‐related” versus “treatment‐unrelated” events and other event subclassifications. While this approach protects against confounding due to lack of blinding, it provides less precise information on the seriousness of adverse events. It should also be noted that heterogeneity bias confidence intervals are frequently wide with low‐event outcomes such as those studied here, and that heterogeneity bias may still be present. Finally, antiplatelet/anticoagulant strategies postdevice implantation varied across studies. While all subjects were exposed to at least 6 months of dual therapy (either full‐dose aspirin and clopidogrel or aspirin and an oral anticoagulant), subjects randomized to Amulet in the Amulet IDE trial were primarily placed on aspirin and clopidogrel while those randomized to Watchman were predominantly given aspirin and warfarin for 45 days following successful implant before transitioning to aspirin and clopidogrel. This postprocedural heterogeneity may at least partially influence relative estimates of safety and effectiveness, but to what extent is unclear. A device comparison trial with homogenous postprocedural exposures would be needed to answer this question.

Abstracting study‐level data can lead to miscoding or misinterpretation of reported outcomes. We minimized this risk by having multiple study members code each article blindly, and by strictly reporting trial‐definition reported outcomes rather than imposing a universal event definition. We also minimized risk of confounding by qualitatively assessing funnel plots for skew and quantitatively assessing risk of heterogeneity bias across trials. While these methods cannot rule out confounding or bias, they suggest that findings are generally homogenous and unlikely to substantially differ with new data. Our findings are substantial enough to warrant long‐term head‐to‐head trials between newer generations of Watchman and Amulet [25].

## 5 Conclusion

In this network meta‐analysis of randomized controlled trials, we found no significant difference in stroke, death, thromboembolism, or device embolization between anticoagulation, the Watchman device, and the Amulet device. However, the findings suggest that the Amulet device may be associated with an increased risk for pericardial effusion, especially when implanted by less experienced operators. Accordingly, LAA occlusion device selection should be based on individual patient characteristics, local practices, and operator experience. Additional studies are needed to further assess the comparative effectiveness of LAAC devices in larger and more diverse cohorts of patients than have been enrolled in the RCTs published to date.

## Conflicts of Interest

John W. Davis has received consulting honoraria from GE Healthcare and Novartis AG for work unrelated to any data or disease processes discussed in this manuscript. Pavel Osmancik has received occasional speaking honoraria from Bayer and Abbott, some of which included discussion of left atrial appendage closure. The other authors declare no conflicts of interest.

## Supporting information

Supporting information.

Supporting information.

## Data Availability

Data will be shared on reasonable request. Analyses, including code for R, are available at rpubs.com for reproducibility. This page is “live” and may be supplemented after study publication.
