# Supplementary material for: Comparing Effectiveness and Safety of Left Atrial Appendage Closure Devices: A Network Meta‐Analysis of Randomized Controlled Trials
Source: Clin Cardiol. 2025 Nov 22;48(11):e70217. doi: 10.1002/clc.70217 (PMC12990298; doi:10.1002/clc.70217)
Supplement: Supplementary file 1 — Supporting information. [file CLC-48-e70217-s001.docx]

# Table of Contents

[Table of Contents 1](#_Toc200207849)

[Search Strategy (Ovid only) 2](#_Toc200207850)

[PRAGUE-17 Trial Data 4](#_Toc200207851)

[**eTable 1. Baseline Characteristics and Risk Factors** 4](#_Toc200207852)

[**eTable 2. Incidence of Composite Primary Endpoint and Its Components in the Presence of Competing Risk** 5](#_Toc200207853)

[Summary of Event Data 6](#_Toc200207854)

[**eTable 3. Trial Event Data** 6](#_Toc200207855)

[eFigure 1. PRISMA Diagram 7](#_Toc200207856)

[Forest Plots 8](#_Toc200207857)

[**eFigure 2. Risk of Stroke** 8](#_Toc200207858)

[**eFigure 3. Risk of Death** 8](#_Toc200207859)

[**eFigure 4. Risk of Embolism** 8](#_Toc200207860)

[**eFigure 5. Risk of Device Embolization** 9](#_Toc200207861)

[**eFigure 6. Risk of Pericardial Effusion** 9](#_Toc200207862)

[eFigure 7. Funnel Plot and Heterogeneity Assessment: Risk of Any Stroke 10](#_Toc200207863)

[Outlier Analysis 10](#_Toc200207864)

[eFigure 8. Funnel Plot and Heterogeneity Assessment: Risk of Death (Any Cause) 11](#_Toc200207865)

[Outlier Analysis 11](#_Toc200207866)

[eFigure 9. Funnel Plot and Heterogeneity Assessment: Risk of Thromboembolism 12](#_Toc200207867)

[eFigure 10. Funnel Plot and Heterogeneity Assessment: Risk of Device Embolization 13](#_Toc200207868)

[eFigure 11. Funnel Plot and Heterogeneity Assessment: Risk of Pericardial Effusion 14](#_Toc200207869)

# Search Strategy (Ovid only)

Ovid Technologies, Inc. Email Service

------------------------------

Search for: 38 not 39

Results: 337

Database: Ovid MEDLINE(R) and Epub Ahead of Print, In-Process, In-Data-Review & Other Non-Indexed Citations and Daily

<1946 to May 30, 2023>

Search Strategy:

--------------------------------------------------------------------------------

1 exp Atrial Fibrillation/ (70532)

2 (atrial adj5 fibrillat*).mp. (104814)

3 (auricular* adj5 fibrillat*).mp. (1591)

4 1 or 2 or 3 (104978)

5 exp Septal Occluder Device/ (3492)

6 amplatzer*.mp. (3353)

7 cardioseal*.mp. (122)

8 septal occluder*.mp. (4216)

9 5 or 6 or 7 or 8 (5842)

10 4 and 9 (935)

11 watchman*.mp. (855)

12 (left atrial adj3 occlusion*).mp. (1409)

13 (left atrial adj3 occlud*).mp. (191)

14 (left atrium adj3 occlusion*).mp. (24)

15 (left atrium adj3 occlud*).mp. (20)

16 (left atrial adj3 closure*).mp. (1573)

17 (left atrium adj3 closure*).mp. (35)

18 11 or 12 or 13 or 14 or 15 or 16 or 17 (3051)

19 4 and 18 (2515)

20 10 or 19 (2772)

21 limit 20 to english language (2639)

22 limit 21 to (systematic reviews pre 2019 or systematic reviews) (175)

23 limit 21 to (meta analysis or "systematic review") (119)

24 22 or 23 (181)

25 limit 24 to yr="2020 -Current" (85)

26 limit 21 to randomized controlled trial (55)

27 exp Placebos/ (39461)

28 random$.tw. (1419026)

29 double blind$.tw. (169904)

30 double dummy.tw. (2471)

31 mask$.tw. (99814)

32 sham.tw. (99861)

33 placebo$.tw. (246272)

34 (control$ adj trial$).mp. (896702)

35 exp Randomized Controlled Trial/ (595179)

36 27 or 28 or 29 or 30 or 31 or 32 or 33 or 34 or 35 (1863754)

37 21 and 36 (356)

38 24 or 37 (441)

39 9 and 18 and 38 (104)

40 38 not 39 (337)

***************************

# PRAGUE-17 Trial Data

**Selection of patients:**

**DOACs:** intention-to-treat population

**LAAC – Amulet:** intention-to-treat population, but limited to successfully performed LAAC procedures

**LAAC – Watchman:** intention-to-treat population, but limited to successfully performed LAAC procedures

## **eTable 1. Baseline Characteristics and Risk Factors**

|  | **DOACs (n = 199)** | **LAAC – Amulet (n = 111)** | **LAAC – Watchman (n = 70)** |
| --- | --- | --- | --- |
| **Demographics** |  |  |  |
| Age (years) | 73.2 ± 7.2 | 72.7 ± 7.1 | 74.5 ± 6.1 |
| <75 years (%) | 122 (60.7%) | 68 (61.3%) | 36 (51.4%) |
| >75 years (%) | 79 (39.3%) | 43 (38.7%) | 34 (48.6%) |
| Male gender (%) | 130 (64.7%) | 78 (70.3%) | 46 (65.7%) |
| Weight (kg) | 88.1 ± 16.2 | 88.0 ± 16.9 | 85.3 ± 18.8 |
| **Clinical history** |  |  |  |
| AF type Paroxysmal (%) | 67 (33.3%) | 28 (25.2%) | 23 (32.9%) |
| Persistent (%) | 46 (22.9%) | 29 (26.1%) | 14 (20.0%) |
| LS persistent (%) | 16 (8.0%) | 9 (8.1%) | 6 (8.6%) |
| Permanent (%) | 72 (35.8%) | 45 (40.5%) | 27 (38.6%) |
| CHA_2_DS_2_-VASc | 4.7 ± 1.5 | 4.7 ± 1.4 | 4.5 ± 1.5 |
| CHA_2_DS_2_-VASc < 3 (%) | 50 (24.9%) | 26 (23.4%) | 19 (27.1%) |
| CHA_2_DS_2_-VASc = 4 (%) | 40 (19.9%) | 25 (22.5%) | 18 (25.7%) |
| CHA_2_DS_2_-VASc = 5 (%) | 57 (28.4%) | 23 (20.7%) | 18 (25.7%) |
| CHA_2_DS_2_-VASc > 6 (%) | 54 (26.9%) | 37 (33.3%) | 15 (21.4%) |
| HAS-BLED | 3.0 ± 0.9 | 3.2 ± 1.0 | 3.0 ± 0.9 |
| Heart failure (%) | 90 (44.8%) | 40 (36.0%) | 36 (51.4%) |
| Hypertension (%) | 186 (92.5%) | 102 (91.9%) | 66 (94.3%) |
| Diabetes mellitus (%) | 90 (44.8%) | 42 (37.8%) | 22 (31.4%) |
| History of cardioembolic event (%) | 69 (34.3%) | 46 (41.4%) | 19 (27.1%) |
| Of which stroke (%) | 63 (31.3%) | 42 (37.8%) | 17 (24.3%) |
| History of MI (%) | 39 (19.4%) | 13 (11.7%) | 12 (17.1%) |
| Randomized at experienced centers | 140 (69.7%) | 60 (54.1%) | 65 (92.9%) |
| **Prior antithrombotic treatment** |  |  |  |
| Warfarin | 104 (51.7%) | 48 (43.2%) | 31 (44.3%) |
| NOACs | 55 (27.4%) | 34 (30.6%) | 23 (32.9%) |
| Aspirin | 32 (15.9%) | 23 (20.7%) | 13 (18.6%) |
| ADP antagonists | 11 (5.5%) | 11 (9.9%) | 2 (2.9%) |
| Dual antiplatelet treatment | 6 (3.0%) | 5 (4.5%) | 1 (1.4%) |
| Other (low dose LMWH, none) | 19 (9.5%) | 12 (10.8%) | 10 (14.3%) |

**DOACs (or NOACs): Direct Oral Anticoagulants (i.e., apixaban). LAAC: Left Atrial Appendage Closure. AF: Atrial Fibrillation. MI: Myocardial Infarction. LS Persistent: Longstanding Persistent. LMWH: Low Molecular Weight Heparin.**

## **eTable 2. Incidence of Composite Primary Endpoint and Its Components in the Presence of Competing Risk**

|  | **DOACs (n = 199)** | | | **LAAC – Amulet (n = 111)** | | | **LAAC – Watchman**  **(n = 70)** | | |
| --- | --- | --- | --- | --- | --- | --- | --- | --- | --- |
|  | **No. of patient with event** | **No. of events** | **Event rate** | **No. of patient with event** | **No. of events** | **Event rate** | **No. of patient with event** | **No. of events** | **Event rate** |
| Primary endpoint | 60 | 81 | 11.92 | 35 | 41 | 10.52 | 10 | 12 | 5.32 |
| Cardiovascular death | 30 | 30 | 4.42 | 15 | 15 | 3.85 | 4 | 4 | 1.77 |
| All-stroke / TIA | 15 | 18 | 2.65 | 12 | 12 | 3.08 | 1 | 1 | 0.44 |
| Ischemic stroke / TIA | 15 | 16 | 2.35 | 11 | 11 | 2.82 | 1 | 1 | 0.44 |
| All-stroke | 11 | 12 | 1.77 | 11 | 11 | 2.82 | 1 | 1 | 0.44 |
| Ischemic stroke | 10 | 10 | 1.47 | 10 | 10 | 2.57 | 1 | 1 | 0.44 |
| Systemic embolism | 1 | 1 | 0.15 | 0 | 0 | 0.00 | 0 | 0 | 0.00 |
| ISTH major/non-major bleeding | 32 | 40 | 5.89 | 17 | 21 | 5.39 | 6 | 7 | 3.10 |
| ISTH major/non-major bleeding not related to device | 32 | 40 | 5.89 | 13 | 17 | 4.36 | 4 | 5 | 2.21 |
| ISTH major bleeding | 19 | 24 | 3.53 | 12 | 14 | 3.59 | 4 | 5 | 2.21 |
| ISTH major bleeding not related to device | 19 | 24 | 3.53 | 10 | 12 | 3.08 | 3 | 4 | 1.77 |
| ISTH non-major bleeding | 16 | 16 | 2.35 | 7 | 7 | 1.80 | 2 | 2 | 0.89 |
| ISTH non-major bleeding not related to device | 16 | 16 | 2.35 | 5 | 5 | 1.28 | 1 | 1 | 0.44 |
| Procedure- or device-related complication | 0 | 0 | 0.00 | 6 | 6 | 1.54 | 2 | 2 | 0.89 |
| Non-cardiovascular death | 23 | 23 | 3.39 | 14 | 14 | 3.59 | 6 | 6 | 2.66 |
| All-cause death | 53 | 53 | 7.80 | 29 | 29 | 7.44 | 10 | 10 | 4.43 |

Event rate is defined as no. of events per 100 patient-years. DOAC: Direct Oral Anticoagulant (i.e., apixaban). LAAC: Left Atrial Appendage Closure. TIA: Transient Ischemic Attack. ISTH: International Society on Thrombosis and Haeomostasis.

# Summary of Event Data

## **eTable 3. Trial Event Data**


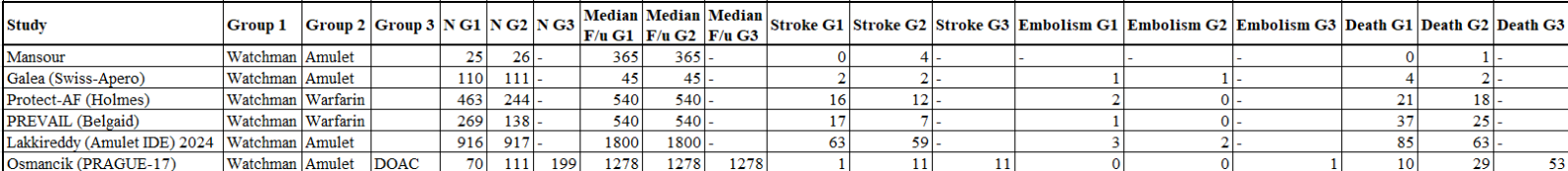


eTable 3 (continued)


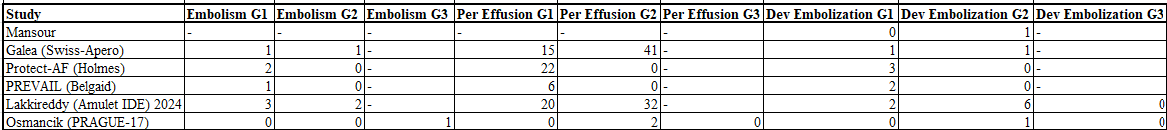


G1: Group 1. G2: Group 2. G3: Group 3. F/u: Follow-up. Per. Effusion: Pericardial Effusion. Dev Embolization: Device Embolization. DOAC: Direct Oral Anticoagulant (i.e., apixaban).

# eFigure 1. PRISMA Diagram

***PRAGUE-17 included after obtaining unpublished, de-aggregated data. Amulet versus Watchman analyses are unrandomized.**

**6* unique, eligible trials identified**

Articles excluded for:

**Not an RCT** (n = 5)

**Ineligible Protocol** (i.e., combined LAAC Devices vs OAC) (n = 5)

**No Eligible Outcomes** (n = 18)

Articles Excluded:

**Wrong Study Design, Mixed Interventions, and/or No Relevant Outcomes Data**

(n = 431)

Articles Excluded:

**Duplicates**

(n = 10)

**Full-text articles assess for eligibility**

(n = 35)

18 Sys. Reviews, Meta-Analyses

13 RCTs

**Total records screened by title and abstract**

(n = 466)

**Records identified through PubMed, Cochrane, Web of Science Databases, ClinicalTrials.gov**

(RCTs, Systematic Reviews, Meta-Analyses)

(n = 476)

# Forest Plots

## **eFigure 2. Risk of Stroke**


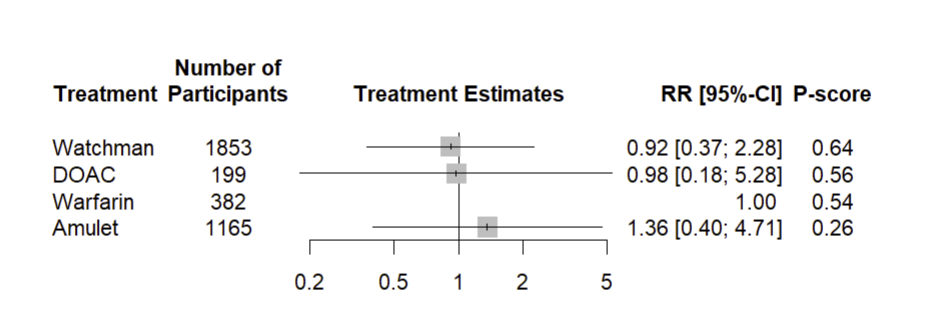


*RR = Risk Ratio, CI = Confidence Interval, DOAC = Direct Oral Anticoagulant (i.e., apixaban)*

## **eFigure 3. Risk of Death**


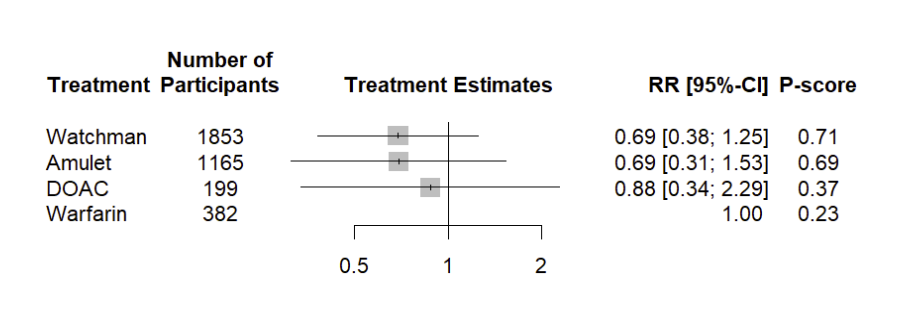


*RR = Risk Ratio, CI = Confidence Interval, DOAC = Direct Oral Anticoagulant (i.e., apixaban)*

## **eFigure 4. Risk of Embolism**


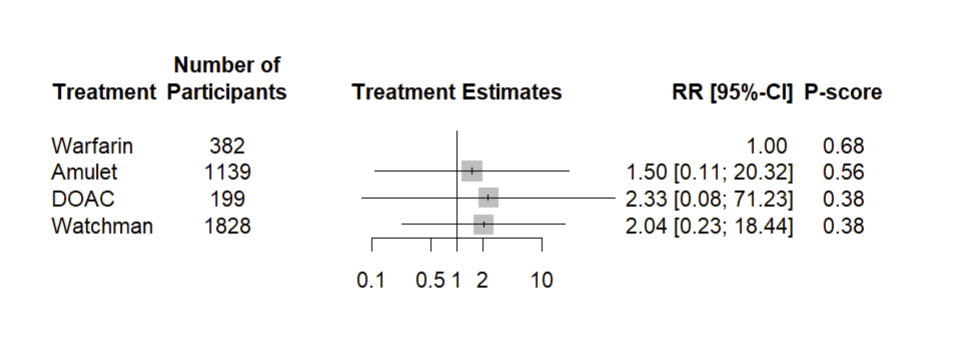


*RR = Risk Ratio, CI = Confidence Interval, DOAC = Direct Oral Anticoagulant (i.e., apixaban)*

## **eFigure 5. Risk of Device Embolization**


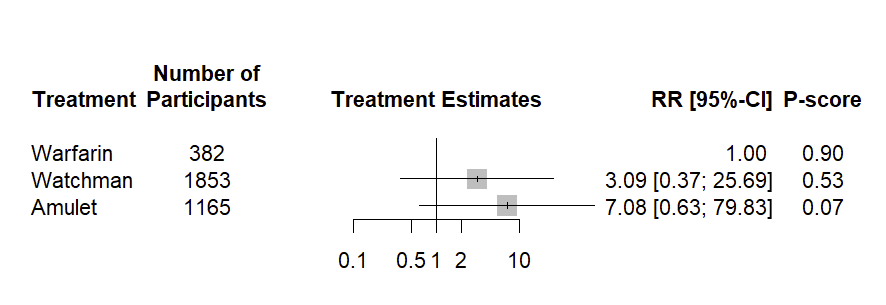


*RR = Risk Ratio, CI = Confidence Interval*

## **eFigure 6. Risk of Pericardial Effusion**


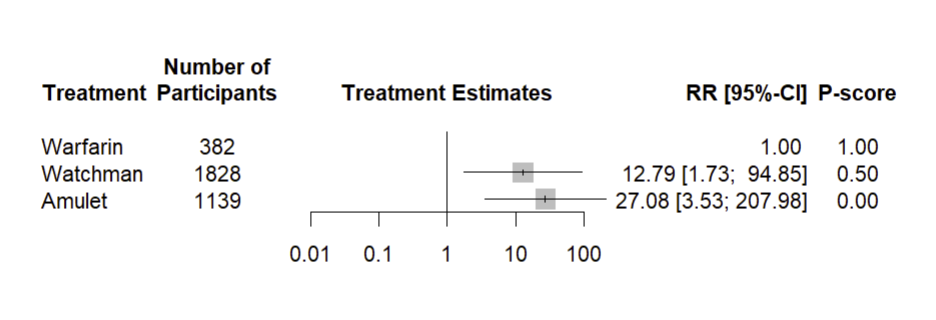


*RR = Risk Ratio, CI = Confidence Interval*

# eFigure 7. Funnel Plot and Heterogeneity Assessment: Risk of Any Stroke


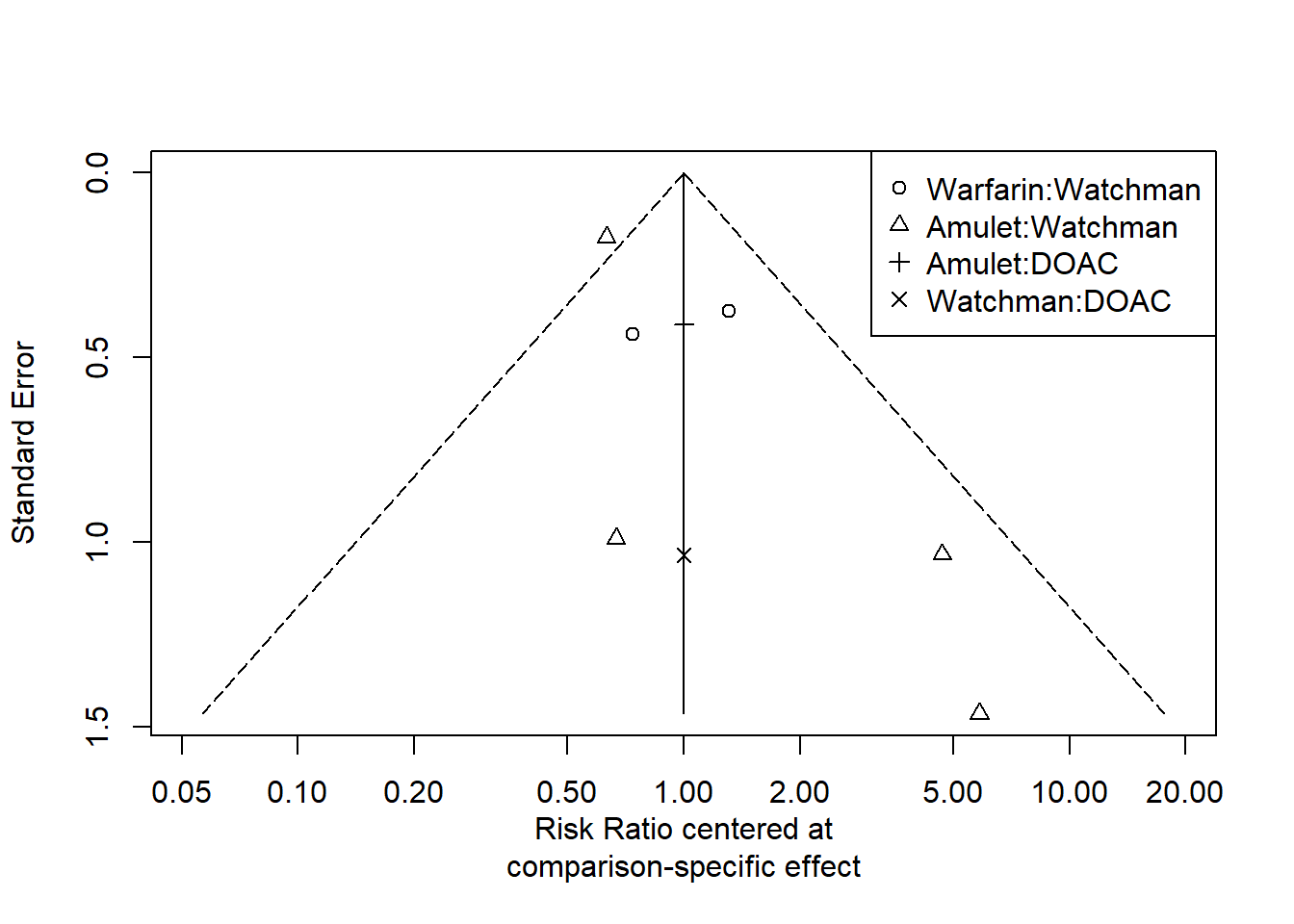


Quantifying heterogeneity / inconsistency:

tau^2 = 0.2636; tau = 0.5135; I^2 = 41.3% [0.0%; 78.4%]

Tests of heterogeneity (within designs) and inconsistency (between designs):

Q d.f. p-value

Total 6.81 4 0.1461

Within designs 3.27 3 0.3519

Between designs 3.54 1 0.0598

### Outlier Analysis

Random effects model

Treatment estimate (sm = 'RR', comparison: other treatments vs 'Watchman'):

RR 95%-CI z p-value

Amulet 3.2154 [0.8856; 11.6748] 1.78 0.0759

DOAC 1.9329 [0.4433; 8.4284] 0.88 0.3804

Warfarin 1.1050 [0.5795; 2.1068] 0.30 0.7618

Watchman . . . .

Quantifying heterogeneity / inconsistency:

tau^2 = 0.0547; tau = 0.2338; I^2 = 12.2% [0.0%; 86.5%]

Tests of heterogeneity (within designs) and inconsistency (between designs):

Q d.f. p-value

Total 3.42 3 0.3319

Within designs 2.50 2 0.2871

Between designs 0.92 1 0.3376

# eFigure 8. Funnel Plot and Heterogeneity Assessment: Risk of Death (Any Cause)


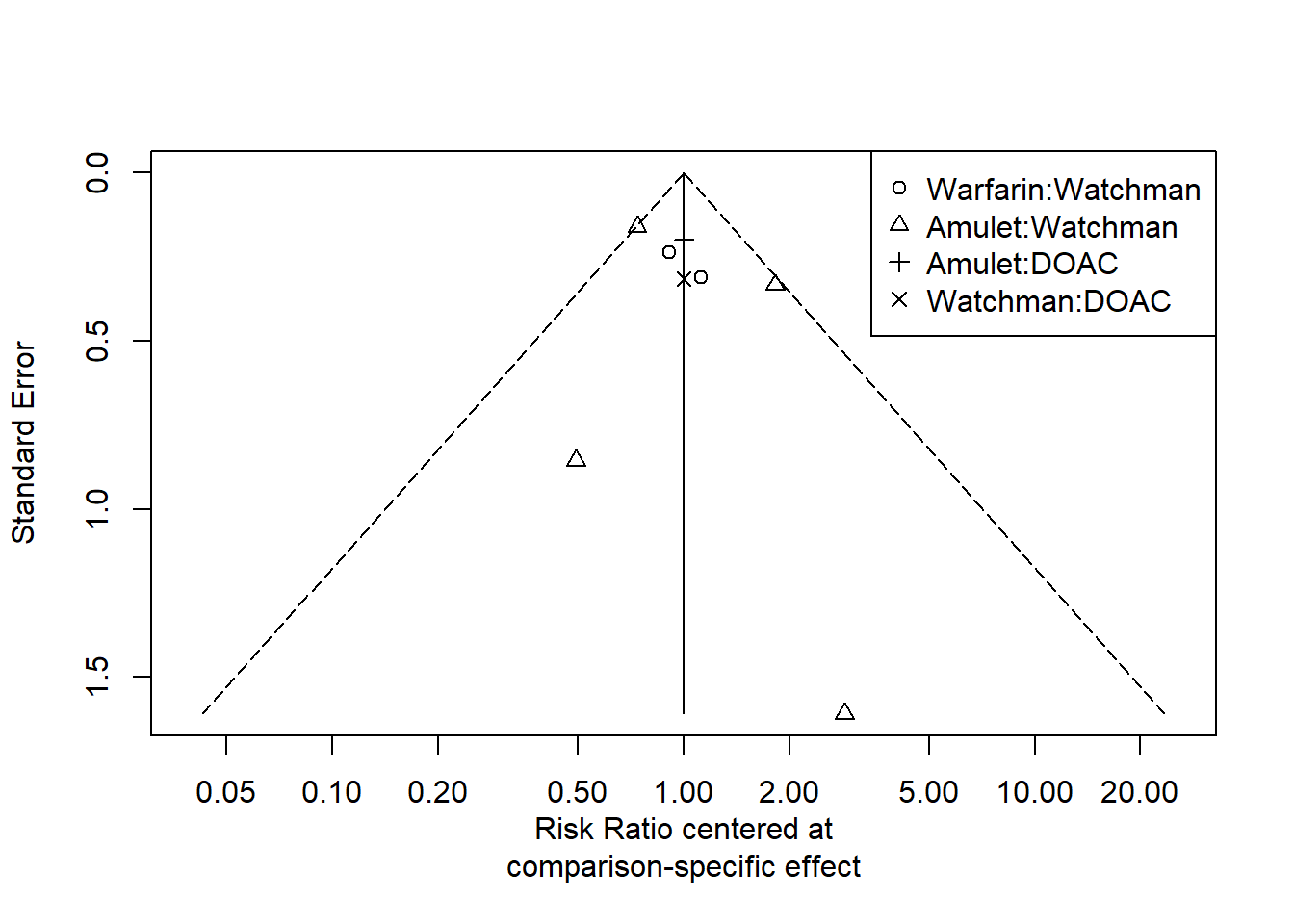


Quantifying heterogeneity / inconsistency:

tau^2 = 0.1067; tau = 0.3267; I^2 = 45% [0.0%; 79.8%]

Tests of heterogeneity (within designs) and inconsistency (between designs):

Q d.f. p-value

Total 7.27 4 0.1225

Within designs 1.23 3 0.7467

Between designs 6.04 1 0.0140

### Outlier Analysis

Random effects model (IDE Exclusion)

Treatment estimate (sm = 'RR', comparison: other treatments vs 'Watchman'):

RR 95%-CI z p-value

Amulet 1.5747 [0.8660; 2.8632] 1.49 0.1366

DOAC 1.6612 [0.9264; 2.9789] 1.70 0.0885

Warfarin 1.4230 [0.9835; 2.0588] 1.87 0.0613

Watchman . . . .

Quantifying heterogeneity / inconsistency:

tau^2 = 0; tau = 0; I^2 = 0% [0.0%; 84.7%]

Tests of heterogeneity (within designs) and inconsistency (between designs):

Q d.f. p-value

Total 2.46 3 0.4824

Within designs 1.23 2 0.5417

Between designs 1.23 1 0.2665

Death NMA P-Score Analysis

P-score

Watchman 0.9523

Warfarin 0.4372

Amulet 0.3549

DOAC 0.2556

# eFigure 9. Funnel Plot and Heterogeneity Assessment: Risk of Thromboembolism

**
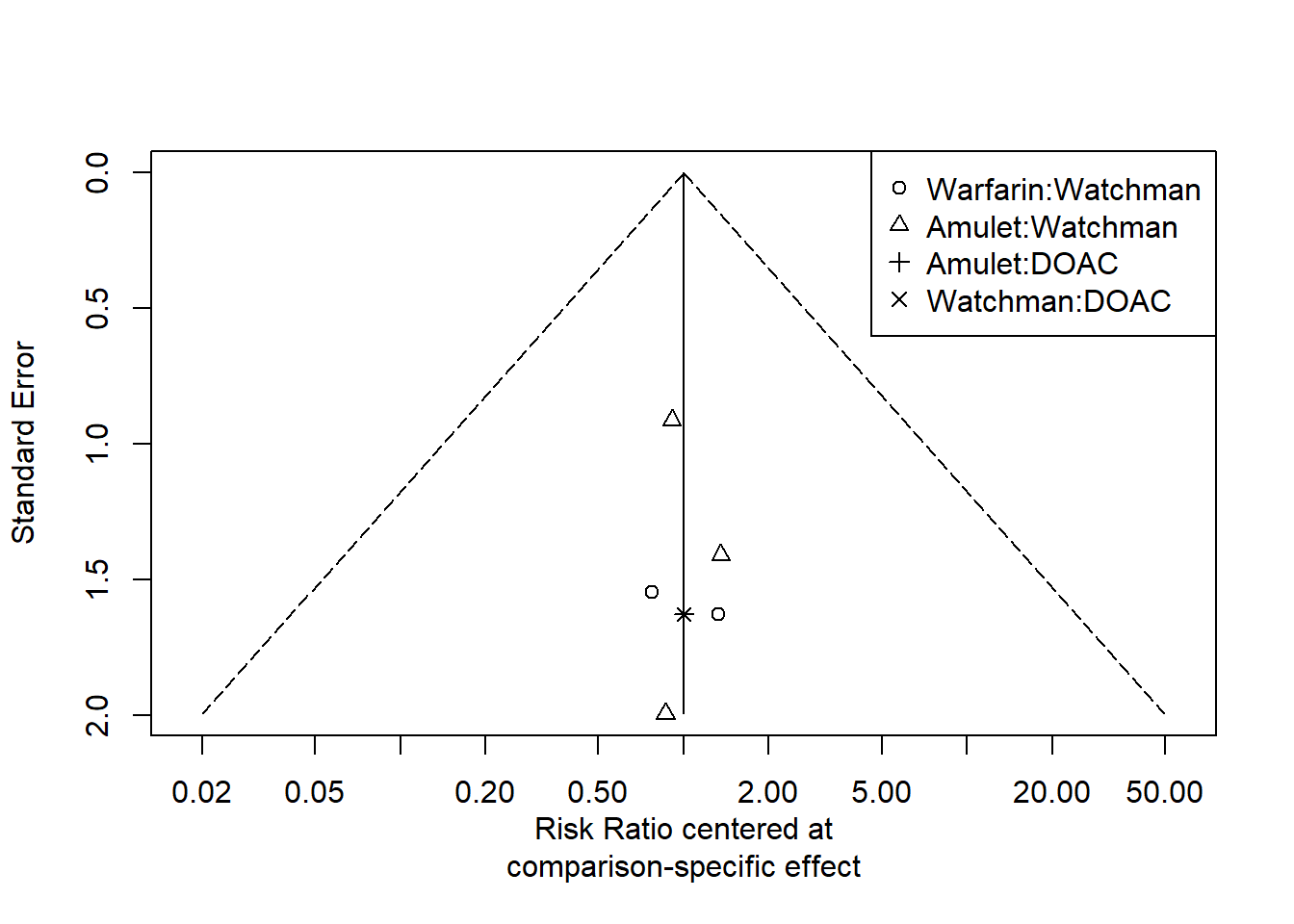
**

Quantifying heterogeneity / inconsistency:

tau^2 = 0; tau = 0; I^2 = 0% [0.0%; 84.7%]

Tests of heterogeneity (within designs) and inconsistency (between designs):

Q d.f. p-value

Total 0.12 3 0.9894

Within designs 0.11 2 0.9449

Between designs 0.01 1 0.9368

# eFigure 10. Funnel Plot and Heterogeneity Assessment: Risk of Device Embolization


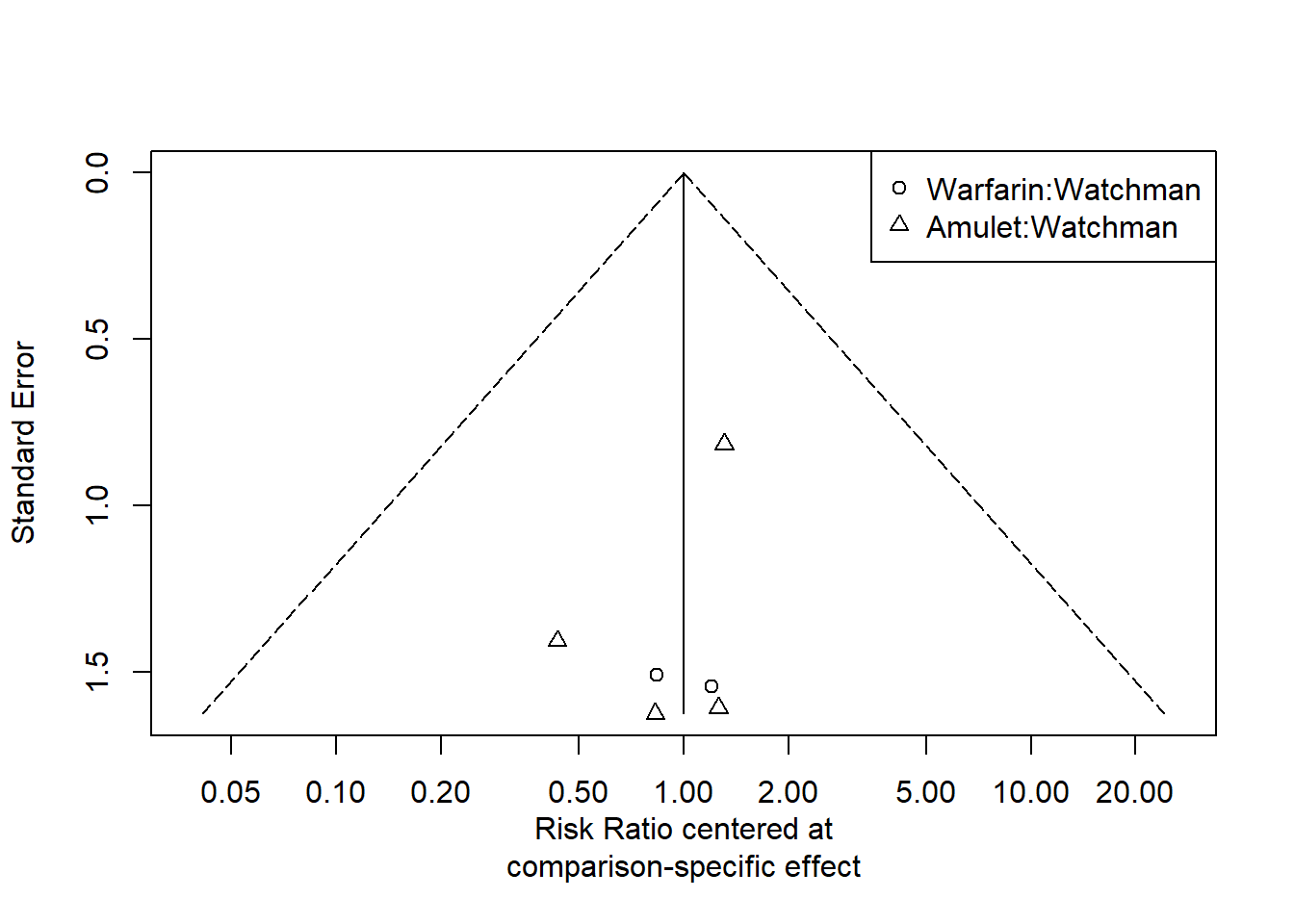


Quantifying heterogeneity / inconsistency:

tau^2 = 0; tau = 0; I^2 = 0% [0.0%; 79.2%]

Tests of heterogeneity (within designs) and inconsistency (between designs):

Q d.f. p-value

Total 0.53 4 0.9710

Within designs 0.53 4 0.9710

Between designs 0.00 0 --

# eFigure 11. Funnel Plot and Heterogeneity Assessment: Risk of Pericardial Effusion


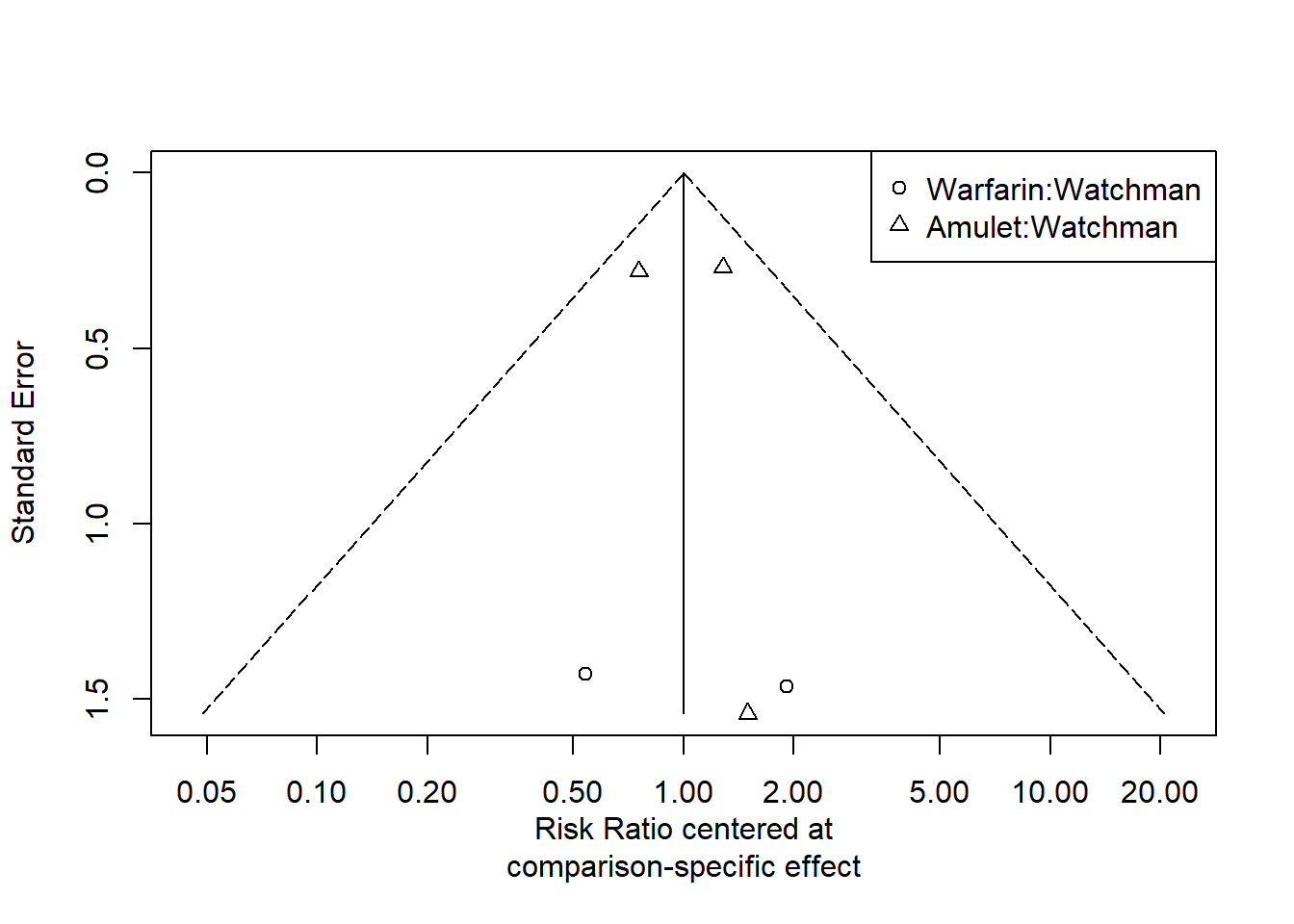


Quantifying heterogeneity / inconsistency:

tau^2 = 0; tau = 0; I^2 = 0% [0.0%; 84.7%]

Tests of heterogeneity (within designs) and inconsistency (between designs):

Q d.f. p-value

Total 2.28 3 0.5156

Within designs 2.28 3 0.5156

Between designs 0.00 0 --
